# Bilateral Mesiodens in Monozygotic Twins: 3D Diagnostic and Management

**DOI:** 10.1155/2013/193614

**Published:** 2013-02-25

**Authors:** Carla Vecchione Gurgel, Ana Lídia Soares Cota, Tatiana Yuriko Kobayashi, Salete Moura Bonifácio Silva, Maria Aparecida Andrade Moreira Machado, Daniela Rios, Daniela Gamba Garib, Thais Marchini Oliveira

**Affiliations:** ^1^Department of Pediatric Dentistry, Orthodontics and Public Health, Bauru School of Dentistry, University of São Paulo, São Paulo, Brazil; ^2^Hospital for the Rehabilitation of Craniofacial Anomalies, University of São Paulo, São Paulo, Brazil

## Abstract

Mesiodens is the most frequent type of supernumerary tooth and may occur in several forms, causing different local disorders, such as impaction of the anterior permanent teeth. High-resolution three-dimensional (3D) images have improved the diagnosis and treatment plan of patients with impacted and supernumerary teeth. The purpose of this paper was to report a case of two mesiodens in monozygotic twin boys with appropriate 3D diagnostic and treatment plan.

## 1. Introduction

A supernumerary tooth is a development anomaly of number characterized by the presence of tooth in addition to the normal series [1]. The mesiodens is the most frequent supernumerary tooth and is located in the maxillary central incisor region [2, 3]. The prevalence of this anomaly varies between 0.15% and 1.9%, being more frequent in males than in females, with a 2 : 1 ratio [3].

 Mesiodens could be discovered accidentally during radiological examination of the premaxillary area. The diagnostic commonly occurs between 7 and 9 years of age probably because permanent central incisors erupt at this stage and the complaint of noneruption induces a radiological examination that might reveal the presence of mesiodens [4]. Several studies have applied cone-beam-computed tomography (CBCT) to accurately diagnose supernumerary teeth with the potential to overcome most of the technical limitations of the plain film projection and the capability of providing a high-resolution three-dimensional (3D) representation of the maxillofacial tissues in a cost- and dose-efficient manner [5–8].

 The occurrence of mesiodens in twins is unusual if not a rare event in the literature. Therefore, the purpose of this paper was to report a case of two mesiodens in monozygotic twin boys with appropriate 3D diagnostic and treatment plan.

## 2. Case Report

 Two monozygotic twin boys were referred to the Pediatric Dentistry Clinic of our University when they were 9 years old for treatment of impacted permanent maxillary central incisors. Their past medical histories showed no systemic diseases, and the dental histories showed no facial trauma or other tooth abnormalities. 

**Figure 1:**
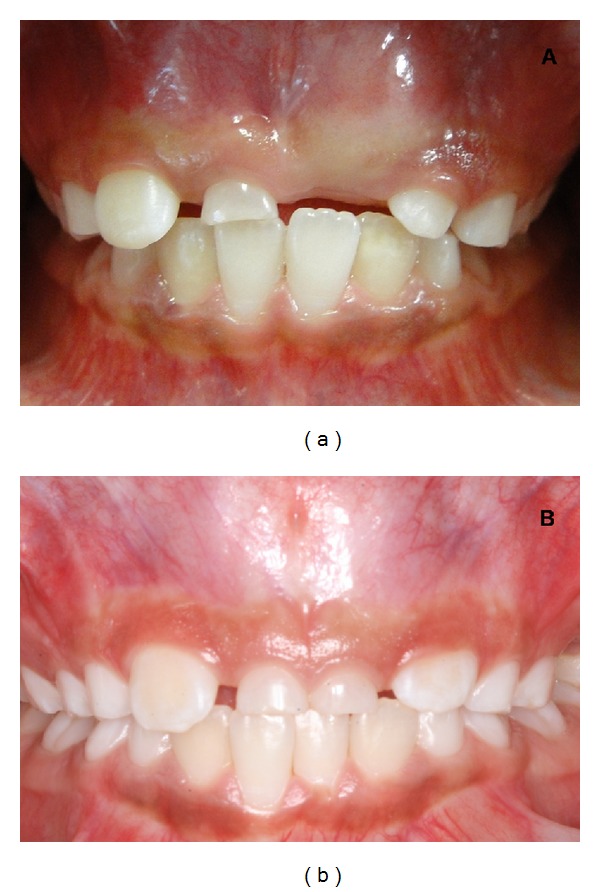
Initial intraoral view showing the absence of the permanent maxillary central incisors in both twins.

 A clinical examination in twin A revealed the absence of the permanent maxillary central incisors and the presence of overretained primary maxillary central right incisor (Figure 1(a)). In twin B the clinical analysis showed the absence of the permanent maxillary central incisors and the presence of overretained primary maxillary central incisors (Figure 1(b)). 

**Figure 2:**
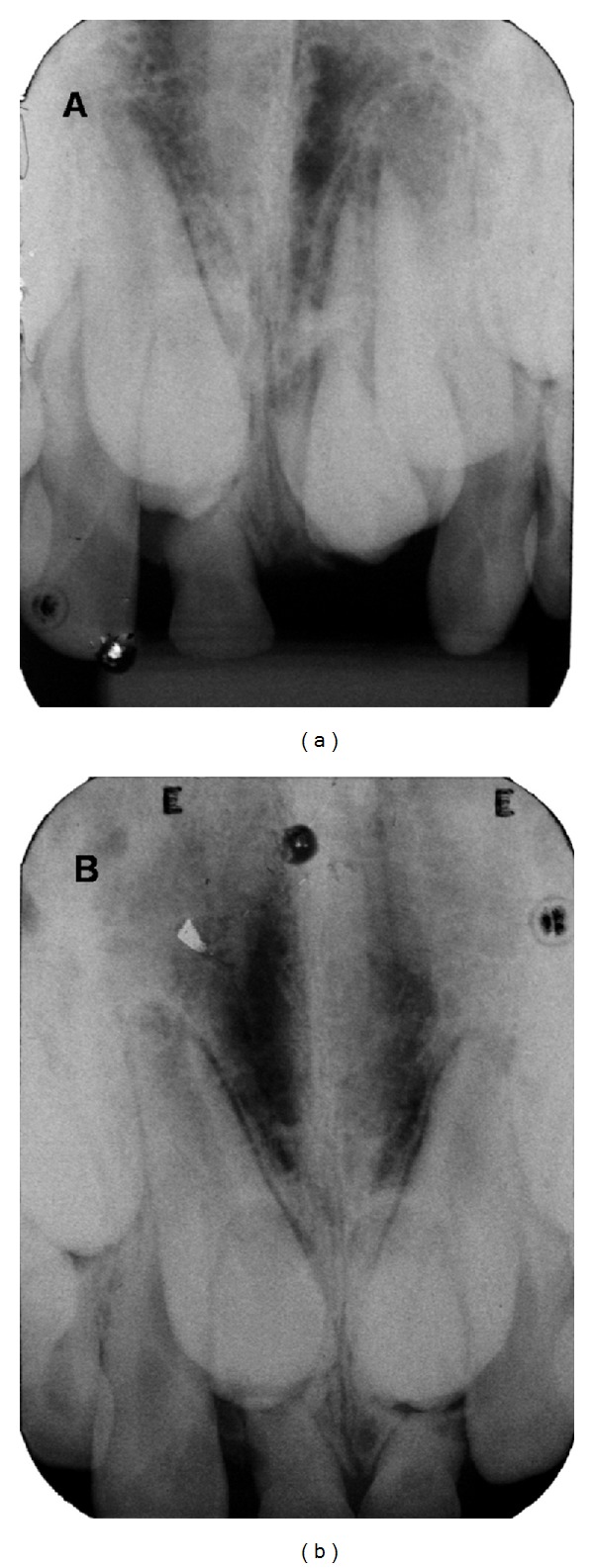
Periapical radiograph showing impacted permanent maxillary central incisors and the presence of two mesiodens in both twins.

**Figure 3:**
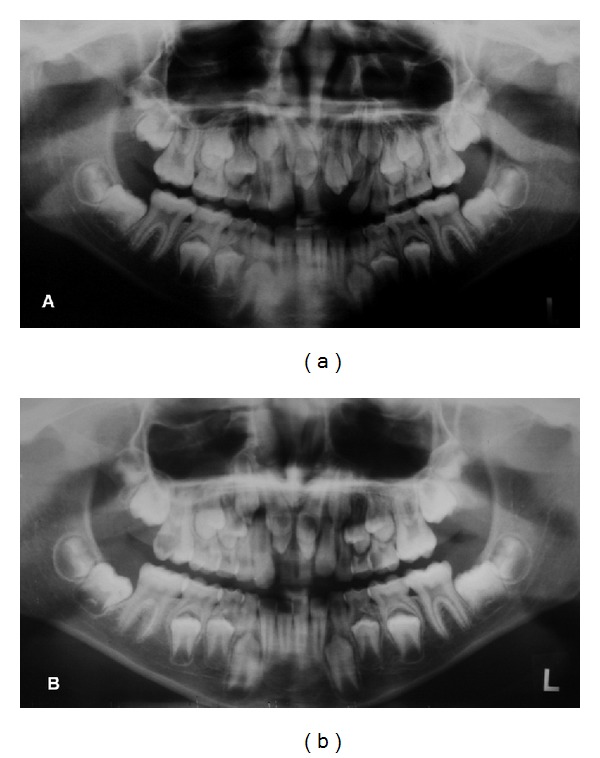
Panoramic radiograph showing impacted permanent maxillary central incisors and the presence of two mesiodens in both twins.

**Figure 4:**
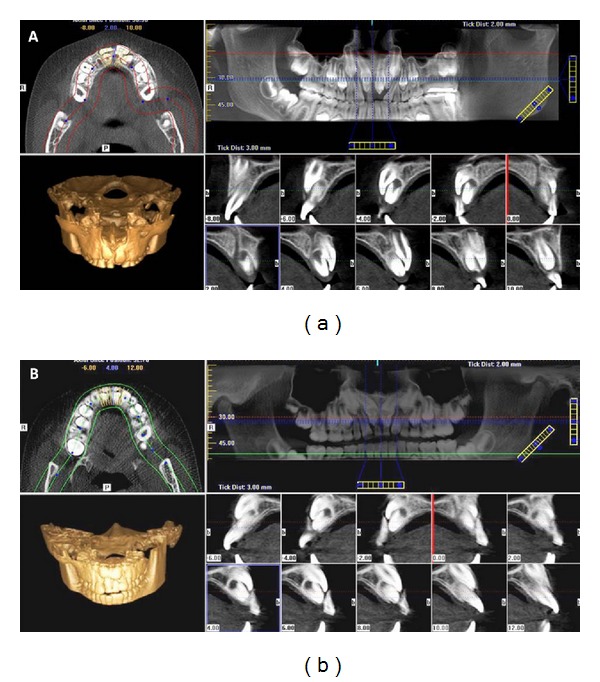
The cross-section oblique images showing impacted permanent maxillary central incisors and the relationship with two mesiodens in both twins.

Panoramic and periapical radiographs were performed in both patients. The radiographs revealed impacted permanent maxillary central incisors because of the presence of two mesiodens in the eruption path in both twins, being that in twin B the supernumerary teeth were in a higher position (Figures 2(a), 2(b), 3(a), and 3(b)). Then, both twins were submitted to CBCT exam of the maxilla to assist in localization and orientation of the two mesiodens. CBCT images were requested for diagnosing accurately the morphology and exact location of the two mesiodens and the radicular formation of the permanent maxillary central incisors. The images were created and viewed interactively using a dental computed tomography software program. Axial sections images revealed horizontal impaction of the permanent maxillary incisors, and cross-section oblique images revealed impacted permanent maxillary central incisors, as well as the relationship with the adjacent teeth and structures (Figures 4(a) and 4(b)).

 After explaining the advantages and disadvantages of the therapeutic options for the patients and their family, the treatment plan was surgical extraction of the two mesiodens and waits for spontaneous eruption of the impacted permanent maxillary central incisors in both twins.

**Figure 5:**
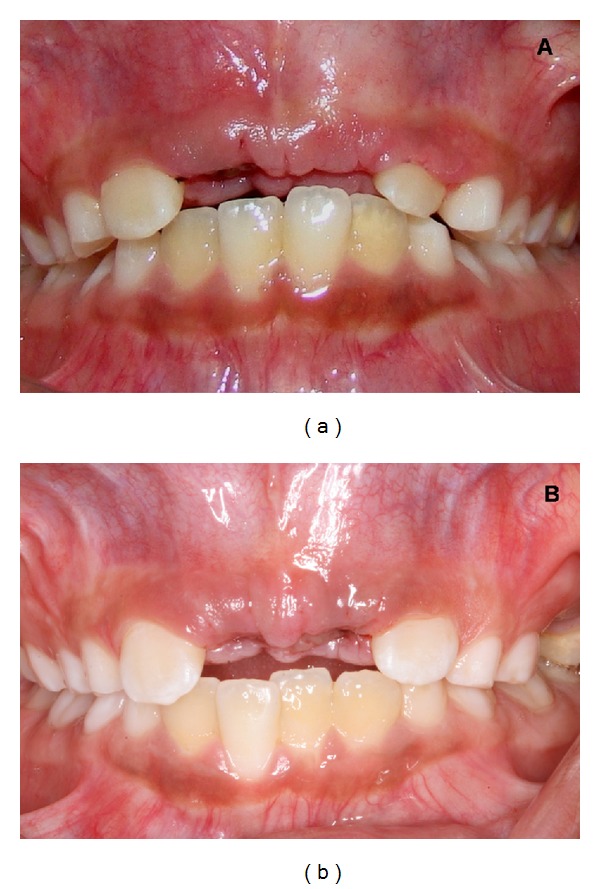
Intraoral view showing the intraoral aspect after 1 week of the mesiodens removal in both twins.

 The surgical technique was performed under local anesthesia. Initially, the overretained primary teeth were extracted. Then, an incision was performed along the gingival margin, from the primary maxillary right canine to the permanent maxillary central left incisor, and a mucoperiosteal flap was elevated to the minimum necessary extent. The mucoperiosteal soft tissues underlying the permanent central incisors were removed. When necessary, the bone which covered the dental crowns was removed with surgical round burs to expose the labial surface. The supernumerary teeth were extracted, and, after cleaning the area and getting hemostasis, the flap was repositioned and sutured. After 1 week, the sutures were removed in both twins (Figures 5(a) and 5(b)).

**Figure 6:**
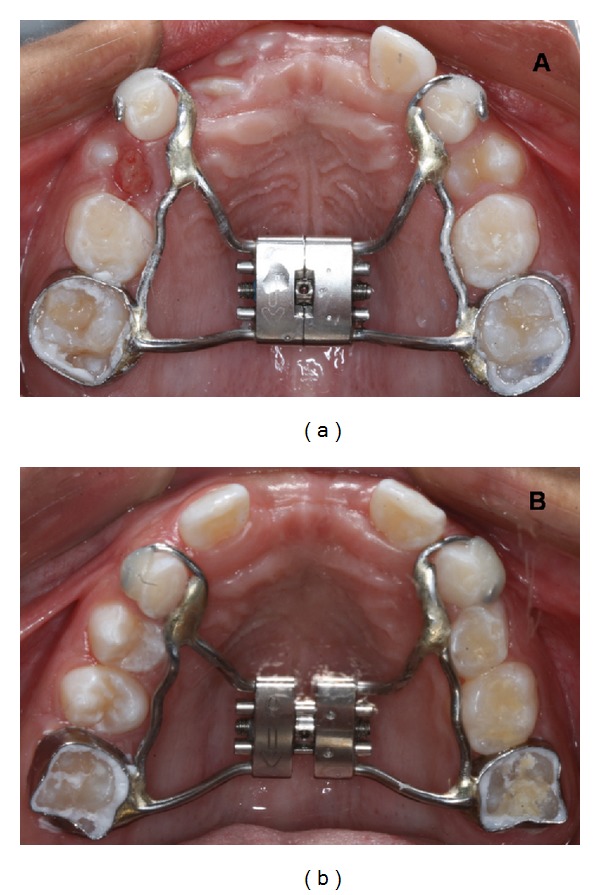
Palatal view of the Hyrax-type palatal expansion appliance in both twins.

**Figure 7:**
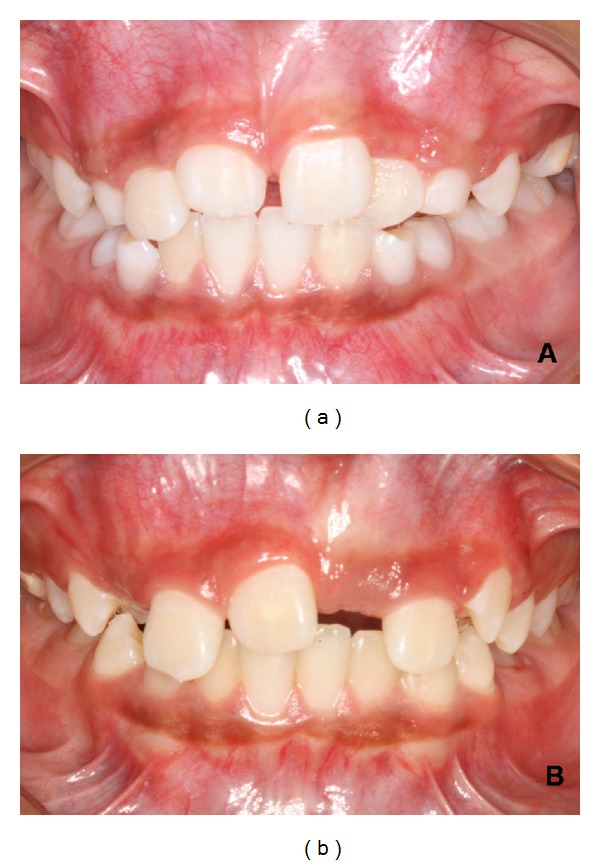
Intraoral view showing the permanent maxillary central incisors erupted in twin A and the permanent maxillary central right incisor erupted in twin B.

 After 4 months of followup, the permanent maxillary central right incisor in twin a erupted in the oral cavity. In both twins, there were a lack of space for the eruption of the permanent maxillary central incisors, and a Hyrax-type palatal expansion appliance was installed (Figures 6(a) and 6(b)). The permanent maxillary central incisors completely erupted in twin A after 10 months of followup (Figure 7(a)). In twin B, the permanent maxillary central right incisor completely erupted after 12 months of the mesiodens removal (Figure 7(b)). However, the permanent maxillary central left incisor had not erupted in twin B after 18 months of followup. The soft tissue, periodontal attachment, gingival contour, and probing depth were normal after eruption in both twins. After 20 months of followup, both twins were referred to orthodontic treatment.

## 3. Discussion

 The etiology of a mesiodens is still not clearly established in the literature. The pathogenesis of mesiodens has been attributed to various theories such as locally induced hyperactivity of the dental lamina, a phylogenetic relic of extinct ancestral tissue, a dichotomy of tooth buds [2, 9], heredity, and some environmental factors [10]. The familial pattern of occurrence of mesiodens in twins strongly supports a genetic influence, possibly inherited as an autosomal dominant inheritance [2, 11, 12]. The theory, involving hyperactivity of the dental lamina, is the most widely supported one. According to this theory, remnants of the dental lamina or palatal offshoots of active dental lamina are induced to develop into an extra tooth bud, which results in a supernumerary tooth [13]. Although no investigation proved the hereditary condition of mesiodens, genetics are also thought to contribute to its development, as such occurrence has been diagnosed in twins, siblings, and sequential generations of a single family [14]. Sedano and Gorlin [12] proposed a genetic theory in which mesiodens is an autosomal dominant trait with lack of penetrance in some generations. A sex-linked pattern has also been proposed, as males are affected twice as frequently as females [15].

The monozygotic twins presented here displayed similarly located supernumerary and impacted teeth suggesting the influence of genetic factors on the etiology of mesiodens. However, some differences observed in the twins dentition suggested that environmental factors may also affect the formation of the phenotype [16, 17]. Seddon et al. [11] concluded, after reviewing eight previous cases and one of their own, that mesiodens were likely to be concordant in monozygotic twins with respect to number, but they noted that minor variations in size, shape, and orientation were common. In the present case, the abovementioned traits are similar in both twins; however, in twin B mesiodens are located in a higher position when compared to twin A. There were some differences during the treatment, but the treatment plan was the same for both twins. Also, in twin A both impacted teeth erupted spontaneously after 10 months while in twin B only the permanent maxillary central right incisor erupted after 12 months of the mesiodens removal. It also suggests an influence of phenotype factors on the occurrence of mesiodens.

The choice of the best treatment plan depends on the correct diagnosis. Oral surgeons require information on both the location and the shape of supernumerary and impacted teeth before performing an operation for extraction. Intraoral and/or panoramic radiography has conventionally been used for preoperative examination [8, 18]. However, panoramic radiography alone is not sufficient for determining the exact location of supernumerary and impacted teeth, due to the image superimposition [6, 19]. CBCT seems to be a good tool for the evaluation, accurate diagnosis, and determination of the location of mesiodens and impacted teeth [5–8]. In the present case, CBCT provided valuable information that helped us to determine the morphology of the mesiodens and exact 3D positioning of the impacted permanent teeth. 

There is no consensus on the literature about the best time for mesiodens removal. Studies have shown that the removal of a mesiodens during the early mixed dentition stage allows normal eruptive forces to promote spontaneous eruption of the impacted tooth after 6 to 24 months [2, 13, 16]. Some authors recommend postponement of surgical intervention until the age of 8–10 years, when unerupted apex of central incisor is almost mature [10]. However, the later the extraction of the mesiodens, the greater the chance that the permanent tooth either will not spontaneously erupt. Unfortunately, by this time the forces that cause normal eruption of the incisors are diminished, and surgical exposure and subsequent orthodontic treatment are more frequently required. Also, space loss and a midline shift of the central incisors may have already occurred by this age, since the lateral incisors will have erupted and may have drifted mesially into the central space. Thus, a significant delay in treatment may create the need for more complex surgical and orthodontic management. In the case presented here, the option was immediately the removal of the mesiodens, and the spontaneous eruption of the maxillary central incisors occurred after 10 months in twin A and 12 months in twin B. However, in Twin B the maxillary central left incisor did not erupt after 18 months of followup, and he was referred to orthodontic treatment. 

## 4. Conclusion

It is necessary to emphasize the role of the dentistry in management of cases of mesiodens, principally, due to the possibility of early detecting of these abnormalities and could establish an adequate treatment plan. Also, CBCT may help in the correct 3D diagnostic and management of impacted and supernumerary teeth.
